# Positive Feeling, Negative Meaning: Visualizing the Mental Representations of In-Group and Out-Group Smiles

**DOI:** 10.1371/journal.pone.0151230

**Published:** 2016-03-10

**Authors:** Andrea Paulus, Michaela Rohr, Ron Dotsch, Dirk Wentura

**Affiliations:** 1 Saarland University, Saarbrücken, Germany; 2 Utrecht University, Utrecht, the Netherlands; 3 Behavioural Science Institute, Radboud University, Nijmegen, the Netherlands; University of Leicester, UNITED KINGDOM

## Abstract

Even though smiles are seen as universal facial expressions, research shows that there exist various kinds of smiles (i.e., affiliative smiles, dominant smiles). Accordingly, we suggest that there also exist various mental representations of smiles. Which representation is employed in cognition may depend on social factors, such as the smiling person’s group membership: Since in-group members are typically seen as more benevolent than out-group members, in-group smiles should be associated with more benevolent social meaning than those conveyed by out-group members. We visualized in-group and out-group smiles with reverse correlation image classification. These visualizations indicated that mental representations of in-group smiles indeed express more benevolent social meaning than those of out-group smiles. The affective meaning of these visualized smiles was not influenced by group membership. Importantly, the effect occurred even though participants were not instructed to attend to the nature of the smile, pointing to an automatic association between group membership and intention.

## Introduction

What does a smile look like? The mental images that come to people’s minds when answering this question probably strongly resemble each other: Certain facial muscle configurations are reliably recognized as smiles, even across different cultures (e.g., [1, 2, 3]). However, recent research shows that there exist subtle differences in the mental representation of smiles (and other emotions) across different cultures ([4, 5]). Moreover, people can discriminate subtle differences within smiles, like, for instance, genuine and deceptive smiles (e.g., [6]), felt, false, and miserable smiles ([7]), and embarrassed, shameful and amused smiles ([8]). It is therefore plausible that the various appearances and interpretations of smiles are reflected in variations in mental representations of a smile (i.e., representations of what, for example, a felt smile or a mischievous smile looks like). However, if this is the case, which of the multitude of representations is activated when people think about a smile?

We argue that depending on situational and social factors, different mental representations of a smile are activated. One promising relevant social factor might be group membership (see also [9]). People tend to evaluate the in-group as positive and the out-group as negative, and this evaluation influences the social relationship between group members ([10–12]). Since different kinds of smiles differ with regard to the meaning they have for the social relationship ([13–16]), one can assume that a factor that influences the social relationship (like group membership) also influences which mental representation of a smile is activated in a given situation: Whereas the mental representation of an in-group member’s smile might consist of an affiliative smile, the mental representation of a smile expressed by an out-group member might consist of a scheming smile or a smile indicating *schadenfreude*. We thus suggest that smiles of in-group and out-group members look different in people’s minds.

Importantly, we argue that these mental representations differ primarily in respect to the communicated social meaning and not necessarily felt affect. Most studies examining differences within smiles focused on genuine versus false smiles, that is, whether the expresser of the smile experienced enjoyment or not. These studies show that humans are able to distinguish between those different kinds of smiles (e.g., [6]), and that smile type influences the evaluation of the expression (e.g., [17]) as well as the evaluation of the expresser (e.g., [14, 15]). However, it has been argued that it is more useful to distinguish the different kinds of smiles based on the function of the smile rather than a separation in genuine and false (see [16], p. 419; [18]). Hess and colleagues, for example, proposed a distinction between smiles that are signs of happiness and those that signal appeasement or dominance ([18]). In a similar vein, Niedenthal and colleagues distinguished enjoyment, affiliative, and dominant smiles ([16]). Considered jointly, these theories propose a distinction between the affective and the social meaning of a smile, that is, whether the smile indicates a feeling or an intention. This distinction relates to the debate about whether emotional expressions express an emotional experience or a social motive ([1, 19]; see also [20]).

Given that the social relationship between expresser and perceiver of an emotion is most important in intergroup situations ([21, 22), it seems plausible that the mental representations of in-group and out-group smiles differ with regard to the signaled social but not necessarily with regard to the affective meaning. Affective meaning (felt affect) and social meaning might be (at least in part) independent of each other: A smile can carry a positive social meaning but the expresser can experience negative affect (e.g., a shameful smile) and vice versa (e.g., mischievous smile). We predict that the activated mental representations of smiles differ between in-group and out-group on the first, but not on the second factor.

Support for the notion that in- and out-group members’ smiles might be conceptualized differently stems from research finding that group membership influences the interpretation of emotional expressions: It has been shown, for example, that even if expresser and perceiver originate from the same culture, out-group emotions are recognized less accurately than those expressed by in-group members ([23, 24]), ruling out differences in the configuration of the expression between groups. Additionally, out-group emotions elicit less facial mimicry ([25, 26]) and less mood contagion ([27]) than in-group emotions. Even more pertinent, Paulus and Wentura ([9]) found that compared to fearful faces, in-group smiling faces elicited relatively more approach behavior, whereas smiling out-group faces elicited relatively more avoidance behavior. Since approach and avoidance reactions are thought to be activated by the social meaning signaled by emotional expressions (e.g., [28, 29]), these findings provide first, but indirect, evidence that in- and out-group smiles are associated with different social meanings. However, there are alternative explanations which can account for these results: It is, for example, conceivable that in-group and out-group emotions are recognized differently or differ with regard to their perceived intensity. A direct test showing that in- and out-group smiles are associated with different social meanings is lacking.

In order to close this gap and provide more direct support for the notion that the mental representations of smiles expressed by in- and out-group members differ with regard to the expressed social meaning, we employed a reverse-correlation image classification technique ([30, 31]). Recently, a number of studies using the reverse-correlation image classification technique have been published in social-cognitive research (e.g., [32–34). In an experiment employing this technique, participants are asked to select one out of two noisy images the one that most likely belongs to a certain category. The presented images consist of a constant base face superimposed with varying random noise patterns. By averaging all images selected for the category of interest, an individual classification image is obtained. The final image constructed with this technique thus represents an estimation of the information diagnostic for this category and can be seen as a visual approximation of a mental representation of the target category ([30, 32]).

The reverse-correlation image classification technique has been used to visualize mental representations of in- and out-group members ([32, 34, 35]), traits ([33, 36]), emotions ([5, 30]), and specific individuals ([30, 37]). Our study visualized the mental representations of smiling in- and out-group members (with group assignment based on a minimal group manipulation) and assessed the different social meanings perceived in these images by participants uninformed about the existence of different groups.

### Overview

We used the reverse-correlation image classification technique to obtain visualizations of what participants think smiling in- and out-group members look like. To this end, after undergoing a minimal group manipulation ([38]; see also [35] for a study combining the reverse-correlation technique with a minimal group manipulation), participants repeatedly indicated which of two noisy smiling faces most resembled a member of either an in-group or an out-group (manipulated between participants). Because we wanted participants to have a mental representation to tap into when completing the reverse-correlation task, we presented photographs of arbitrary, neutral-looking men and women as in- and out-group members prior to the reverse-correlation task in a minimal group-paradigm. Each photograph was a morph between a unique individual and the in-group or out-group “prototype” (i.e., pictures of two other specific individuals). As a result, the images belonging to a single group had a modest “family resemblance”. This procedure was employed to facilitate building up a mental representation about the respective groups.

All images used for the reverse-correlation task consisted of a constant base image of a smiling male face, with a random noise pattern superimposed. The noise pattern altered the features as well as the expression of the base face differently on each trial. The emotional expression was not mentioned in the instructions. The noise pattern of the images selected in the classification task were averaged per participant in order to obtain the personal classification image of a smiling in- or out-group member, representing a visualization of what that participant thought a typical smiling in- or out-group member looked like.

After the image creation phase, a second sample of participants—blind to the manipulation—rated each of the obtained personal classification images on various variables related to the perceived kind of smile. These variables were selected to assess the meaning of smile, that is, its expressed social meaning (i.e., benevolent vs. malevolent) as well as its underlying affect (positive vs. negative). We hypothesized that the mental representations of in-group and out-group smiles should differ with regard to expressed social meaning but not perceived affect. Therefore, group membership of the images on which the classification image was based should be the best predictor for the social meaning signaled by the smile. By contrast, attributed felt affect should be unrelated to the group membership of the images on which the classification image was based. Finally, participants rated the clarity of the picture, intensity of the emotional expression, and intelligence of the depicted person to control for potentially moderating variables.

In addition to being rated, the obtained individual classification images were coded with the Facial Action Coding System (FACS; [39, 40]). The FACS coding objectively quantified the expression-related differences between the obtained classification images.

## Method

### Ethics statement

All individuals taking part in the study provided written informed consent for participation. The study (including the consent procedure) was approved by the Ethics Committee of the Faculty 5 Empirical Social Sciences of Saarland University.

### Image Creation

#### Participants

Forty non-psychology students (23 Females, 17 Males) at Saarland University, Germany, participated in the image creation phase of the study. This phase lasted approximately 40 minutes. Participants were paid 6 Euros for compensation.

#### Design

The experiment followed a one-factorial design with two conditions (group membership: in-group vs. out-group) varied between participants.

#### Materials

For the minimal group manipulation we presented two groups of photographs of arbitrarily selected Caucasian men and women as in- and out-group members to participants. The two groups consisted of six morphs each, which were created by morphing the photograph of each of six people with the photograph of a seventh person (the morphing factor was 50%). In doing so, the six members of a given group resembled each other to a degree that was noticeable but not blatant. The photographs were taken from the Radboud Faces Database ([41]), the Amsterdam Dynamic Facial Expression Set ([42]), and our own collection ([43]) and displayed men and women with neutral facial expressions.

In the main part of the experiment, participants categorized 800 pictures that were presented as pairs side by side. Each picture consisted of the base face with a random noise pattern superimposed. The random noise pattern was created by summing truncated sinusoid images in six orientations (0°, 30°, 60°, 90°, 120°, and 150°), five spatial frequencies (2, 4, 8, 16, and 32 patches per image, with each patch spanning two sinus cycles), and two phases (0, p/2), with random contrasts (amplitudes).

**Fig 1:**
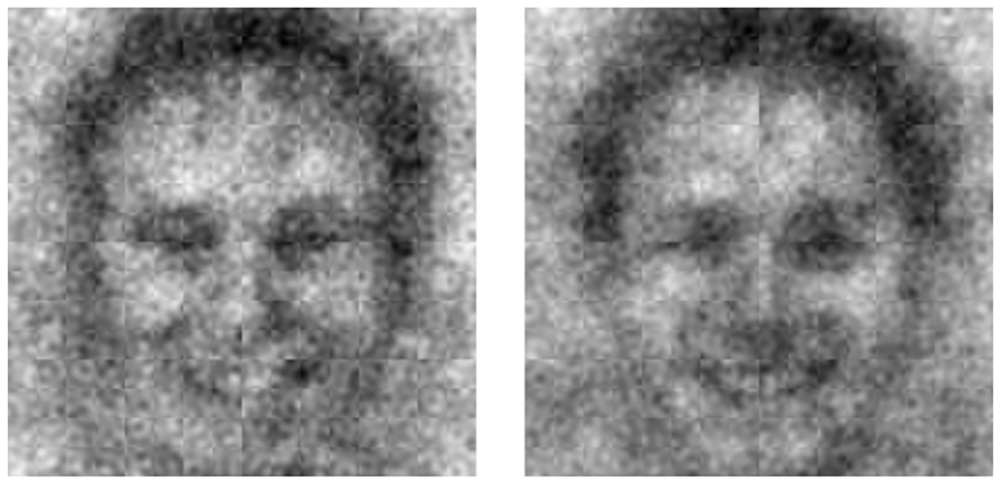
Example of a stimulus pair presented in the classification task. Participants were instructed to select the image that resembled a member of the *focal* group.

The base face was the male smiling face of the Averaged Karolinska Directed Emotional Faces Database ([44]). Each pair consisted of the base image with one particular noise pattern superimposed and the base image with the negative of this noise pattern superimposed (i.e., originally dark pixels were bright and vice versa; see also [32]). Since the base face expressed a smile, all images showed a smiling expression (see Fig 1 for an example of the stimuli used in the experiment).

#### Procedure

Participants were seated individually in front of personal computers, separated by partition walls. Before the main part of the experiment, the reverse correlation image classification task, a minimal group manipulation was conducted. The minimal group manipulation consisted of an ostensible perception style test and a learning phase, designed to create a mental representation of the respective in- and out-group. All instructions were given on the computer screen.

At the start of the group manipulation, participants were informed that the first part of the experimental session would be an assessment of their perception style. Then the ostensible test of perception style was started. In this perception test (taken from [45]) participants indicated what they perceived in ambiguous pictures (mostly taken from [46]). After the rating of eleven pictures, participants received ostensible feedback (unrelated to their actual responses in the test), telling them that they possessed a *basal* perception perceptual style or a *focal* perception style. Participants were informed that people having a *basal* perception style processed stimuli in their environment starting from the background whereas people having a *focal* perception style processed stimuli in their environment starting from the salient features. Assignment to perception style was randomized.

After the perception test, the cover story told participants that the goal of the main experiment was to examine how the individual perception style influences the recognition and the categorization of other people with the same or a different perception style. They were then shown the two groups of morphed faces with first names. They were told that the members of one group possessed a *basal* perception style, and the others possessed a *focal* one. To enhance the manipulation, within the group of people that had the same perception style as the participant, a silhouette of a male or female face with the label “Me” was embedded. (To parallelize, within the other group, a silhouette with a randomly chosen first name was included. The silhouette representing the participant was that of a face of the same sex as the participant whereas the other silhouette was that of a face of the opposite sex.) After familiarizing the faces, participants completed at least 56 trials in which they had to categorize these pictures as in- and out-group members. The learning phase terminated the earliest after 56 trials or after the participant had categorized each of the faces consecutively without making a single error. It was implemented so that participants would create a mental image about the in- and out-group on which they could base their decision in the image creation task. The assignment of groups of photographs to the *basal* and *focal* group was held constant throughout the experiment. However, since perception style was varied between participants, each group of photographs was the in-group for half of the participants and the out-group for the other half.

After participants had successfully learned the categorization of the morphed faces, they were told that the goal of the next part of the experiment was to assess how the individual perception style influences the categorization of noisy faces. Then the main part of the experiment, the reverse correlation image classification task started. Participants were shown the pairs of noisy images side by side (see Fig 1 for an example) and were asked to select the image that most resembled a member of the *focal* group. Thus, half of the participants always selected the image that resembled an in-group member and half selected the image that resembled an out-group member. Note that all participants could refer during the reverse-correlation task to a mental image that was based on exactly the same exemplars shown during learning. Participants completed 400 trials. After each block of 80 trials, the participants were reminded to always select the pictures that resembled a member of the focal group and the overview of the focal group was presented again. Even though all noisy images showed a smile, this expression was not explicitly mentioned to participants.

After finishing the entire experiment participants were thanked and dismissed. They were completely debriefed via email after the completion of the data collection.

The noise patterns of the 400 images that each participant had selected in the reverse correlation task were later averaged per participant and superimposed on the base image to create the classification images. Thus, we obtained 40 classification images (one per participant), 20 visualizing the mental representation of smiling in-group member and 20 visualizing that of a smiling out-group member.

### Image rating

The meaning of the displayed smile of the 40 obtained images (i.e., 20 images created on the basis of the categorizations of those participants who were instructed to select out-group members and 20 images of participants who were instructed to select in-group members) were rated by an independent group of participants. Furthermore, we collected ratings on three control variables (see below).

#### Participants

Twenty-nine psychology students (20 females, 9 males) at Saarland University, Germany, rated the smiles. A further 30 psychology-students (18 females, 12 males) rated the faces on the control variables. The ratings lasted approximately 30 minutes and were part of a one-hour experimental session. Participants were paid 8 Euros for participation.

#### Procedure

We gave descriptions of seven possible meanings of a smile to the raters and asked them to indicate how much each face expressed each of these meanings. We chose these meanings based on the literature concerning different types of smiles ([13, 16]) and on our own considerations. Descriptions of these meanings are displayed in Table 1. Please note that these meanings contain—to different degrees—information about the social as well as the affective meaning of the emotion; a single meaning can thereby contain information about the social as well as the affective meaning of the smile. As was already argued above, social and affective meaning might be affected to a different extent by the manipulation of group membership.

**Table 1:** Meanings of Smiles Items.

Item	*The smile shows…*
Intrinsic Smile	*… that the person is happy about something that is not related to the presence of another person*.
Social Smile	*… that the person is happy about the presence of another person*
Shameful smile	*… that the person is ashamed about something*
Superior smile	*… that the person feels superior*.
Mischievous smile	*… that the person is happy about the misfortune of another person (Schadenfreude)*.
Scheming smile	*… that the person has dishonest intentions*.
Polite smile	*… that the person is just smiling because it is expected in the particular situation*.

Before the start of the rating procedure, information about the different meanings was given and participants rated two images (created by two student research assistants with the same reverse-correlation procedure) to familiarize themselves with the procedure. The classification images obtained in the image creating phase were then presented in random order. Participants indicated on scales ranging from 1 (not at all) to 10 (very much) to which extent the smile signaled each of the seven meanings (see Table 1). The rating scales for each meaning were presented below each other on the computer monitor. Thus, each face was rated on each of the seven meanings before the next face was presented. The ratings showed a high internal consistency across the raters with ICCs ranging from .79 to .94 (we employed a two-way random model with measures of consistency for calculating ICCs.).

To obtain indicators of discriminant validity, a further sample of independent participants rated (a) the intelligence of the displayed person, (b) the clarity of the image, and (c) the intensity of the emotion. We collected these ratings to be able to control for perceptual differences in the appearance of the pictures and differences in the perceived intensity of the expression. By collecting ratings for a positive trait not associated with emotion or group membership (i.e., intelligence), we were able to assess if group membership specifically influences the mental representation of smiles or if the mental representations between in-group and out-group members differ in general valence (which potentially influences the ratings of the smiles).

These ratings were collected independently from the emotion rating to prevent influences of the prior rating of emotion meaning. Intelligence, image clarity, and emotion intensity were collected in consecutive blocks, that is, each of the 40 classification images were rated on one variable before the next variable was assessed. Intelligence was rated on a scale ranging from 1 (stupid) to 10 (smart). Clarity and intensity were rated on a scale ranging from 1 (very low) to 10 (very high). As with the emotion meaning ratings, participants completed two practice trials before they rated the classification images. The raters showed satisfactory interrater reliability (Intelligence: ICC = .92; Clarity: ICC = .94; Intensity: ICC = .96. Again, we employed a two-way random model with measures of consistency for calculating ICCs.).

The obtained individual classification images were coded by two certified coders according to the Facial Action Coding System (FACS; [39, 40]), in order to objectively capture differences between the facial expressions shown in the images. The FACS coders were blind to classification image condition and to the study’s topic and hypotheses. To assess general reliability of the coder ratings, the coders were given fourteen additional photographs of individuals depicting prototypical emotional expressions (i.e. joy, anger, fear, neutral, sadness, disgust, surprise, depicted by a male and female person). The two coders showed good reliability on these control images (FACS index of agreement = .88; see [47] for a calculation of the FACS index of agreement). The FACS index of agreement for the classification images was .74 between the raters, indicating moderate but satisfactory reliability. Thus, the two coders were able to assess deviations in facial appearance resulting from facial muscle movements in a valid and reliable way. (To pass the FACS final test for being certified as a professional FACS coder, a human coder must reach an agreement of.70 with the criterion coding of the test. Excellent coders achieve an agreement of .80 and above. Note, that these evaluations are related to coding of natural faces. Our coders reached an agreement of .88 for the natural faces, indicating that they were excellent coders).

## Results

### Image Rating Task

The ratings for each of the seven meanings of a smile, as well as the clarity, intensity, and intelligence ratings were averaged across raters. This resulted in one score for each of these variables for each classification image. The mean values, as well as tests of the simple in-group out-group differences for the seven meaning variables for the in-group and out-group condition are displayed in Table 2. The mean values for clarity, intensity, and intelligence are reported S1 Table.

**Table 2:** Mean values as well as well as t-values and p-levels of the simple comparisons between the in-group and out-group condition for the Meaning of Smiles Items (standard deviations in parentheses).

Item	In-group (SD)	Out-group (SD)	*t*-Value	*p*-level
Intrinsic smile	4.70 (.83)	3.96 (1.08)	2.45	.019
Social smile	5.84 (1.22)	4.31 (1.81)	3.15	.003
Shameful smile	2.87 (.98)	2.72 (.83)	.55	.586
Superior smile	2.62 (.55)	3.47 (1.43)	-2.47	.018
Mischievous smile	3.05 (.89)	4.21 (1.77)	-2.62	.013
Scheming smile	2.45 (.69)	4.05 (1.96)	-3.45	.001
Polite smile	4.89 (.84)	4.34 (1.41)	2.62	.141

*Note*: Ratings had to be given on a scale ranging from 1 (not at all) to 10 (very much).

We report the non-adjusted, exact *p*-values here. They should be understood as descriptive rather than definite signs of significance (see [62]).

Since it is likely that the meaning rating scales contain (to varying degrees) information about the social meaning as well as the affective state of the perceiver, we submitted the seven meaning variables for each image as data points to a principal component analysis in order to test the hypothesized structure. As predicted, this resulted in a two factorial solution with the first factor explaining 67% of the variance and the second one explaining 24%. (With regard to some “rules of thumb” for performing PCA [or factor analysis], the reader might wonder about the ratio of N [40] to number of items [7]. Note that those “rules of thumb” [e.g., 10:1] usually refer to data sets of raw ratings [i.e.,”noisy” data]. Here, each data point that enter into the PCA is an aggregate of N = 29 raw ratings. Thus, on the level to which the “rules of thumb” apply, we effectively have a [40 x 29 =] 1160:7 = 166:1 ratio.)

The two factors with the respective communalities and loadings, after VARIMAX-rotation, of the seven variables are displayed in Table 3 (comparable results emerged if we employed a principal axis factoring or an oblique rotation.). As can be seen, the variables that were mainly descriptive for the social meaning signaled by a smile showed high loadings on the first factor; meanings describing a negative social meaning thereby showed negative loadings (mischievous, superior, scheming) whereas meanings describing a positive social meaning showed positive loadings (social, polite). We therefore interpreted the first factor as representing the social meaning (benevolent vs. malevolent) signaled by a smile. The second factor, in contrast, was interpreted as indicative for the affective meaning of a smile since the meanings describing positive affect showed high positive loadings (social, intrinsic) and the meaning describing negative affect had a negatively signed loading (shameful). Since we believe that most (if not all) items entail a social and an affective meaning to a certain degree, we extracted factor score variables, allowing each item to be part of both factors. The assumption that the same item can entail both meanings is nicely demonstrated by the item “shameful”: The loadings of the item “shameful”, which were substantial on both factors, had a positive sign for factor one and a negative sign for factor two, indicating the presence of positive social meaning and the absence of positive affect. A higher value on the first factor (social meaning) indicated more benevolent social meaning whereas a higher value on the second one (affective meaning) indicated more positive affect.

**Table 3:** Communalities and Factor Loadings for the Meanings of Smiles Items (PCA; VARIMAX rotation).

Item	Communality	Factor 1 (social meaning)	Factor 2 (affective meaning)
Intrinsic smile	0.92	.52	.**81**
Social smile	0.95	**.64**	**.73**
Shameful smile	0.92	.43	**-.85**
Superior smile	0.93	**-.95**	.16
Mischievous smile	0.93	**-.96**	-.07
Scheming smile	0.97	**-.93**	-.32
Polite smile	0.81	**.90**	-.07

*Note*. Loadings > .60 (absolute values) are printed in bold.

In order to assess the influence of group membership, intensity of the emotion, clarity of the expression, and intelligence of the expresser on the two respective factors, we conducted hierarchical multiple regression analyses on both factor scores separately (the simple correlations of the control variables with group membership and the two factors are reported in S2 Table). As already presented in the introduction, we hypothesized that group membership would be the best predictor for the social meaning signaled by a smile. The affective meaning behind the smile, however, should not be predicted by group membership (but presumably rather by intensity). We entered group membership as the first predictor in the regression analysis because this allowed us to assess its influence on the two factors throughout every step of the analysis. Furthermore, it was the only factor which was experimentally manipulated (e.g., [48]). Please remember that a hierarchical regression analysis is nothing but a successive application of multiple regression analyses with one, two, three, and four predictors; each single step of a regression analysis is not influenced by the order of the entrance of the predictors. Therefore, the result of the final step of our analysis is identical to the result obtained with a hierarchical regression analysis in which group membership were entered last.

**Fig 2:**
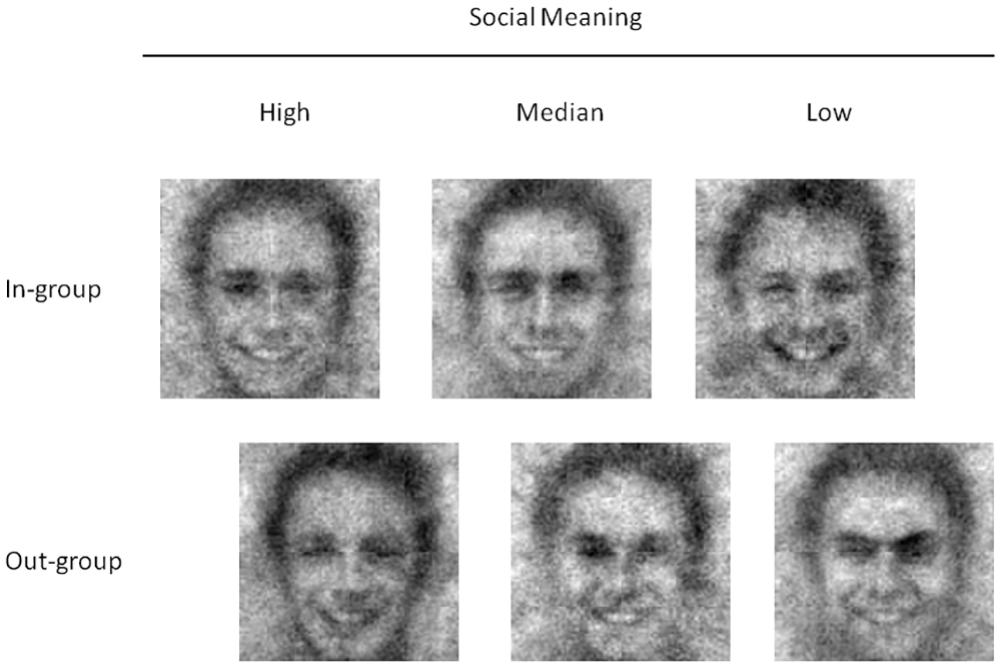
Examples of classification images obtained in the experiment with regard to the factor social meaning. The upper row displays images of in-group members, the lower row images of out-group members. The left column displays the image with the highest score on the factor social meaning (for the respective group), the middle column displays the image with the median score with respect to the distribution of the factor social meaning (for the respective group), and the right column displays the image with the lowest score on the factor social meaning (for the respective group). A higher score indicates more benevolent social meaning. The rows displaying images of in-group and out-group members are shifted to indicate the correlation between group and the factor social meaning.

As can be seen in Table 4, the results supported our hypotheses. Group membership (out-group = 0, in-group = 1) was a significant predictor of the factor social meaning in Step 1 and remained a significant predictor after intensity was entered in Step 2, clarity in Step 3, and intelligence in Step 4. The signs of the beta weights show that in-group smiles were associated with a more positive social meaning than out-group smiles (see Fig 2 for images for the in- and out-group selected on the basis of the social meaning). Intensity, which was clearly a non-significant predictor of the factor social meaning in Step 2, surprisingly reached significance in Step 3 and Step 4. However, we believe that this change in significance is due to a suppression effect caused by the correlation between clarity and intensity (intensity and clarity as well as clarity and social meaning were significantly correlated, *r* = .55 and *r* = .52, respectively, intensity and social meaning did not significantly correlate, *r* = .14). Clarity was a significant predictor when entered in Step 3, but failed to reach significance when intelligence was entered in Step 4. Intelligence did not significantly explain additional variance (i.e., the change in *R*^2^ was not significant for Step 4). Therefore we base our main interpretation on Step 3 of the analysis. Importantly, the results of every step show that group membership significantly predicted social meaning above and independently of the other variables throughout the analysis. The results therefore support our hypothesis that group membership influences the social meaning signaled by a smile.

**Table 4:** Results of the Hierarchical Regression Analysis (Beta-Weights and R^2^).

	Factor 1 (positive social meaning)				Factor 2 (positive affective meaning)			
	Step1	Step2	Step3	Step4	Step1	Step2	Step3	Step4
*Predictor*								
Group	.40**	.39**	.38**	.32*	.19	-.04	-.04	-.04
Intensity		.01	-.34*	-.32		.69**	.72**	.73**
Clarity			.63**	.45			-.07	-.09
Intelligence				.22				.02
*R*^2^	.16*	.16*	.43**	.44**	.04	.45**	.46**	.46**

*Note*. Group: out-group = 0, in-group = 1

** indicates *p* < .01

* indicates *p* < .05 (one-tailed for Group, two-tailed otherwise; exact *p*-values for Group were *p* = .006, *p* = .009, *p* = .004, and *p* = .026 for Steps 1 to 4, respectively

The same analysis was also run on the factor affective meaning. The results show that intensity was the only significant predictor (see Table 4). Group membership never predicted the affect shown in a smile, even if it was the only predictor. These results support our hypothesis that in- and out-group membership is not associated with the affective component of the smile. The affect shown in a smile is only inferred from the intensity of the expression.

### FACS coding

In order to reduce complexity of the FACS results and to quantify possible differences between the pictures regarding the expressed smiles, we created three new variables based on classification of Action Units: presence of AU12 (smiling mouth), presence of AU6 (eye wrinkles), and presence of smile-atypical AUs (all AUs except AU 6, 7, 12, 13, and AUs related to mouth opening, i.e., 25, 26. AU 7 (narrowed eye aperture due to lid tightening) was not regarded as atypical for smiling since it can occur in intense smiles, but it was not regarded as typical as it is not seen as a specific indicator for smiling. The same applies to AU 13 (sharp lip puller) which can also occur in smiling. AUs 25 and 26, indicating mouth opening, were also disregarded, because mouth opening is a typical consequence of an intense AU 12, but not specific for smiling.) These three variables allowed us to assess if the differences in the ratings between in-group and out-group smiles arose because in-group images displayed typical smiles more often, if they arose because out-group images displayed smile-atypical facial muscle changes more often, or both. The variables indicating presence of eye or mouth muscle activity were coded as frequency variables, that is, each picture was scored with 1, if the respective AU was coded by both coders (indicating clear presence of the AU), and with 0 if the AU was not coded or only coded by one coder (indicating no clear presence). The variable indicating atypical AUs was created by summing the number of coded atypical AUs (i.e., if a picture was coded with 6+10+12+25 by one coder, and 4+6+12+25 by the other coder, it received a score of 2 since AU 6 and 12 were seen as typical for a smile and AU 25 was disregarded (see also footnote 9)). Of all mentioned atypical AUs, AU 10 (upper lip raiser), related to contempt, was mentioned most often (17.5%), followed by AU 4 (brow lowerer; 12.5%), which is typically seen in anger. Other mentioned AUs were AU 14 (lip dimpler), related to contempt, AU 15 (lip corner depressor), observed in sadness, AU 20 (lip stretcher) and AU 5 (upper lid raiser), which are often observed in fear, AU 29 (jaw thrust), AU 1 (inner brow raiser), AU 2 (outer brow raiser) and AU 5 (upper lid raiser).

To statistically test for differences between in-group and out-group in the variables related to muscle activity around the mouth and eyes, we conducted two logistic regressions with group as dependent variable and the eye or mouth variable as predictor. The logistic regression with presence of smiling mouth as predictor yielded no significant effect, *b* = 21.31, *z* = 0.00, *p* = .999. All except two of the classification images were rated as containing AU 12. Entering presence of eye wrinkles as predictor yielded a significant effect, *b* = 1.24, *z* = 1.87, *p* = .031 (one-tailed). AU 6 was coded more often in in-group (65%) than in out-group (35%) images. Numerically, atypical AUs were observed more often for out-group (Sum = 17) than for in-group images (Sum = 11); the distributions in the two groups did, however, not differ significantly, Mann–Whitney *U* = 171.00, *p =* .445.

## Discussion

The results of our experiment support the hypothesis that the mental representation of smiles expressed by in-group and out-group members conveys different social meanings. By employing the reverse-correlation image classification technique we visualized participants’ mental representations of smiling in-group and out-group members. Independent participants then rated the meaning of the smile shown by these images. In addition, we captured the differences in facial configuration of the images via FACS coding.

The results of the participants’ ratings demonstrate that the social meaning signaled by the respective smile differed as a function of group membership: As predicted, the mental representations of in-group smiles expressed a more benevolent social meaning than those of out-group smiles. Importantly, the mental representations of in- and out-group members’ smiles did not differ in underlying affect but only in communicated social meaning. As argued above, the felt affect and communicative signal should be (partly) independent. The results indeed showed that group membership was a significant predictor of the social meaning of the smile but not of the affective meaning. Intensity was the only predictor of the underlying affect of the visualized smile. The more intense the expression in an image was rated, the more the smile expressed positive affect. This effect was independent of group membership.

The result of the FACS coding shows that AU 6, which causes wrinkles around the eyes when smiling, was coded more often in in-group images compared to out-group images. There was, however, no difference regarding the smiling mouth or the atypical AUs. These results show that the mental representation of in-group smiles, visualized as classification images, approached a typical smile more often than the mental representation of out-group smiles. These results furthermore provide first evidence regarding the perceptual features characterizing benevolent and malevolent smiles.

The results of our study provide important insight into the mental representation of smiles as well as the association between group membership and social meaning. We will discuss them in turn.

The result that group membership predicted social meaning, but not affect shown in a smile, is an important finding since it shows that the results obtained in our experiment cannot be explained by a simple match of the valence associated with the respective groups and the valence of the smiles: One might argue that participants always selected the face showing the more positive smile as an in-group member because the evaluation of this expression more closely matches the evaluation of the expresser. This interpretation is supported by research showing that an association between the emotional expression and the evaluative connotation of the group membership of the expresser of an emotion facilitates emotion recognition ([49–51]). The results of our experiment, however, point to a specific association between group membership and social meaning: The mental representations of the smiles expressed by positively evaluated in-group members did not express more positive affect but only more benevolent social meaning, if the two factors are separated. Thus, group membership does not bias the mental representations of a smile towards general positivity or negativity but only towards specific positive or negative characteristics (see also [33], for a related finding).

Importantly, the effect occurred even though participants in the reverse correlation task were not instructed to base their decision on the nature of the smile but rather on arbitrary group images. The emotional expression was neither task-relevant nor mentioned. However, the resulting in- and out-group images show that the smiles influenced participants’ categorizations. Therefore, we assume that this influence occurred spontaneously and probably even unintentionally; it is caused by an association between group membership and specific social meanings of a shown facial expression. This association influences the visualized mental representation of group members which in turn influences the decision. This conclusion is supported by a recent study published by Tskhay and Rule [52]: The authors demonstrated that the mental representations of perceptually ambiguous groups (i.e., groups based on political affiliation or sexual orientation) are characterized by specific emotional expressions. Comparably to our study, participants completed a reverse correlation task in which they selected the noisy image resembling the member of a specific group. Even though emotional expression was not mentioned, the resulting classification displayed different facial expressions. Although the focus of this study differed from ours (i.e., it examined the association between certain groups and certain emotional expressions, not between group membership and subtle differences within the same expression), the results support the notion of an automatic association between group membership and certain emotional expressions.

These results of the subjective image rating task are substantiated via FACS coding. Eye wrinkles, which are seen as indicators for genuine smiles ([53]), were coded more often for in-group images than for out-group images; this pattern of coding indicates that participants in the rating task used specific differences related to smiling to make their responses. Specifically, one might assume that participants inferred felt affect mostly based on a smiling mouth (AU 12), for which we observed no differences, and used the presence of eye wrinkles (AU 6) to infer social meaning. Although speculative, this is an interesting result since eye wrinkles are usually seen as indicators for genuine smiles ([54]), that is, indicators for felt affect. However, there is also results showing that smiles with eye wrinkles are also judged as being more interpersonally positive and elicit more affiliative responses ([55, 56). Furthermore, there is evidence that these cues cannot reliably differentiate between spontaneous and posed smiles ([57]). Based on these findings one might therefore speculate that the way specific facial expressions are used might depend on the specific task context given. In our study, participants were asked to rate the smiles for quite specific meanings. Given this task, they might have attended to the configuration of typical and atypical indicators of smiles to come to a conclusion. For the same reason, our result might be a consequence of the specific “noisy” appearance of the employed static picture. In both cases, our results might not generalize fully to all other contexts of smiling.

The finding that group membership influences the social meaning associated with an emotion also shed light on the results of studies examining reactions to in-group and out-group emotions. The (relatively) negative reactions to smiles expressed by out-group members have often been explained as caused by the negative social meaning seen in those images ([9, 26]). However, these results could also be explained by a simple match between the valence of the expresser and the valence of the emotion: Research has shown that an expression was rated as more intense if the evaluation of the group membership of the expresser and the evaluation of the emotional expression matched ([50]). However, our results suggest that group membership influences exclusively the social and not the affective meaning of a smile. Since mental representations reflect previous experiences as well as expectancies ([4]), it is therefore plausible to assume that the different reactions to in-group and out-group smiles arise because group membership influences the interpretation of the social meaning of a smile.

Finally, our results are also of importance for studies examining the question of how individuals recognize the different meanings of smiles and other emotional expressions: There is some debate about whether the meaning of an expression is communicated and decoded through specific configurations of the expression or through situational factors (e.g., [13, 16, 58–61]). Whereas the first view assumes that there exist different kinds of smiles which can be recognized without contextual information, the latter states that there exist no prototypical exemplars of different smiles. Shedding some light into this debate, our results show that different meanings of a smile are (at least in part) associated with specific characteristic features of the expression and that the perceivers (the raters in our experiment) can distinguish between those meanings even if no situational information is provided.

Despite providing many answers, our study also poses new questions. One question concerns the role of the clarity of the judged images: as the results of our study show that, in addition to group membership, clarity was also a significant predictor of the social meaning: Clearer expressions were rated as expressing more benevolent social meaning. This shows that perceptual features of smiles indeed influence the interpretation of the meaning. We think the finding that clarity of the expression predicts the rating of social meaning makes perfectly sense: The perceiver has to be able to decode the social meaning in order to recognize it as benevolent. If the social meaning cannot be decoded because the expression is less clear, the perceiver might feel uncertain, rating the social meaning as less benevolent. This finding can be connected to theories assuming an influence of imitating behavior on the interpretation of smiles ([16, 17]): A blurry, unclear expression cannot easily be imitated; accordingly, the interpretation of the smile might be guided by conceptual knowledge. However, we think that the results regarding clarity have to be treated with some caution: Group membership was the only factor manipulated during the image creation task; therefore only the results regarding this factor allow us to draw inferences about *mental representations* of smiles.

Another potential caveat is related to our decision to make the emotion not task-relevant: One might argue that we actually visualized in-group and out-group *faces* instead of in-group and out-group *smiles*. However, we think this is rather unlikely: First of all, we found quite specific differences between in-group and out-group smiles. In our view, such a result was very unlikely if participants had used permanent facial characteristics pertaining to in-group and out-group members to resolve the task. We believe that in this case, the resulting images would have mainly differed with regard to person characteristics and not with regard to characteristics about smiles. Thus we conclude that participants relied on different mental images of the smiles when solving the task. Second, since all the images in the categorization task showed a smile, it seems plausible that participants imagined a smiling in-group or out-group member to handle the task. However, future studies should empirically address this point.

A final point concerns the ecological validity of our approach. The base face employed in our study was an averaged face created from posed facial expressions. One might argue that this image does not show a realistic smile, diminishing the validity of our results. However, we do not believe the unrealistic nature of the base face poses a problem since research employing the reverse correlation technique shows that the choice of the base face does not constrain the final classification image as much as one might think: First, recent reverse correlation work on facial expressions using dynamic stimuli (Jack et al. 2012) presented computer generated video clips of faces with random facial movements. The resulting classification movie clips looked highly plausible and realistic, even though the original stimuli judged by participants made many implausible movements. Second, previous research using exactly the same reverse correlation methodology and a base face from the same database (i.e., an average Scandinavian face) as our study produced valid Moroccan and Chinese looking faces (e.g., [32, 33]). The fact that a Scandinavian face can be used as basis for plausible Moroccan and Chinese classification images demonstrates the advantages of the reverse correlation technique: The superimposed noise changes facial appearance in such a way that many interpretations can be made on the faces (i.e., almost any plausible judgment seems possible with reverse correlation). The method thus creates the opportunity to “shape” the resulting images according to features contained in the mental representation which are not necessarily included in the base face. Thus, we think that our approach provides evidence for different mental representation of in-group and out-group smiles which should also be relevant in more natural settings.

A sign for ecological validity of our results is provided by the FACS codings. The results showed that perceivers could reliably detect such perceptual features in the images, which are also observed to be important for smiling in natural images. One might object that the coding results did not provide strong evidence regarding the perceptual features differentiating between in-group and out-group smiles. We agree that the statistical evidence is relatively weak, since only one of the three variables we used to quantify the features of the smiles differentiated between in-group and out-group images. However, we believe that the reason for this can be found in the nature of the images: The classification images created with the reverse correlation task were quite noisy, as can be seen in Fig 2. Assessing specific muscle activations, as it is done with FACS, is therefore quite difficult. As a result, the coders might have hesitated in attributing a specific Action Unit to a picture, in order not to interpret “noise” as “signal”. Indeed, according to the FACS manual a specific Action Unit should only be scored if at least a trace of appearance change due to muscle movements is clearly visible. Thus, the codings can only be seen as a rough estimation of the actual perceptual features associated with in-group and out-group smiles. Nevertheless, the codings provide first evidence regarding the perceptual features associated with in-group and out-group smiles.

Finally, one might object that several of the items describing the smiles showed high loadings on the factor social meaning as well as the factor affective meaning. As explained shortly in the introduction, this is not unexpected: Since most (if not all) of the employed meaning items encompass to varying degrees social as well as affective meaning aspects, this is also reflected in the factor loadings. The item “shameful” illustrates this nicely, as already described above. However, the same reasoning also holds for the other items such as, for example, the item “social smile”. We believe that a “social smile” indicates that the person is affiliatively smiling at somebody else (e.g., greeting that person), but possibly also shows that she/he is experiencing a positive affect about the presence of the other person. For the items which show lower cross-loadings, the meaning regarding felt affect might simply be less important for the perceiver and therefore be represented in the factor felt affect to a lesser degree. Moreover, our task did not directly target different types of smiles, but rather attributions which people make to in-group and out-group smiling faces constructed using reverse correlation. Thus, the stimulus images are noisy and constructed, so that they do not provide information about only one specific kind of smile. Given the variety of situations in which smiles can be encountered, this seems very plausible to us—in the image construction phase, no information about the intergroup situation (i.e., if it was cooperative or competitive, hostile, openly aggressive) was given. Also, there may have been variation across participants in the image construction phase regarding which smile they associated with in-group or out-group status. Our result does, however, not put into question that different kinds of smiles (or specific mental representations for different kind of smiles) exist. Our research suggests that in-group and out-group mental representations are associated with (several) types of smiles, which can be classified as conveying varying degrees of felt affect or intention. This results might also be due to the way we created group membership (i.e., with the minimal group paradigm), thereby priming a vague or general imagination of an out-group. Using a specific out-group with specific associated stereotypes (e.g., pre-activating knowledge about Arabs as proud people, or Chinese as very polite, but not openly showing negative intentions) could perhaps influence specific types of smiles only. This issue was, however, lies beyond the scope of the present work. It might, however, be interesting to follow up on the exact interplay of felt affect and social meaning of different kinds of smiles in future research. Our approach might provide a promising method to do this.

Taken together, the results of this experiment show that social factors influence the mental representation of an emotional expression: The mental representations of in-group members’ smiles showed more benevolent social meanings than that of out-group members. Interestingly, the mental representations of in- and out-group smiles did not differ in expressed affect. This effect points to an association between group membership and social meaning signaled by an emotional expression, and not between group membership and general valence.

## Supporting Information

S1 Table Mean values of the control variables, separately for in-group and for out-group. (DOCX)

S2 Table Simple correlations of the factor group, the control variables, and both factor scores. (DOCX)
