# Supplementary material for: Positive Feeling, Negative Meaning: Visualizing the Mental Representations of In-Group and Out-Group Smiles
Source: PLoS One. 2016 Mar 10;11(3):e0151230. doi: 10.1371/journal.pone.0151230 (PMC4786158; doi:10.1371/journal.pone.0151230)
Supplement: S1 Table — (DOCX) [file pone.0151230.s001.docx]

**S1 Table. Mean values of the control variables, separately for in-group and for out-group**

| Item | In-group | (SD) | Out-group | (SD) |
| --- | --- | --- | --- | --- |
| Clarity | 3.41 | (.84) | 3.00 | (1.10) |
| Intensity | 5.74 | (.95) | 4.87 | (1.50) |
| Intelligence | 5.61 | (.86) | 4.67 | (1.13) |

*Note:* Ratings had to be given on a scale ranging from 1 (not at all) to 10 (very much).
