# Supplementary material for: Positive Feeling, Negative Meaning: Visualizing the Mental Representations of In-Group and Out-Group Smiles
Source: PLoS One. 2016 Mar 10;11(3):e0151230. doi: 10.1371/journal.pone.0151230 (PMC4786158; doi:10.1371/journal.pone.0151230)
Supplement: S2 Table — (DOCX) [file pone.0151230.s002.docx]

**S2 Table. Simple correlations of the factor group, the control variables, and both factor scores**

| Item | Intensity | Intelligence | Group | Factor1 | Factor2 |
| --- | --- | --- | --- | --- | --- |
| Clarity | .55** | .85** | .21 | .52** | .32* |
| Intensity |  | .48** | .34* | .14 | .67** |
| Intelligence |  |  | .44** | .58** | .28 |

*Note:* ** indicates *p* < .01, * indicates *p* < .05 (two-tailed)
